# 

**Published:** 1889-09

**Authors:** 


### Bristol Medico-Chirurgical Journal.

### SEPTEMBER, 1889.

### CONTENTS.

ORIGINAL PAPERS: *Page*

Reminiscences of an Army Surgeon. By WILLIAM JOHNSTONE FYFFE, M.D. ... ... ... ... ... ... ... ... ... ... ... ... ... ... 145

Accidental Hæmorrhage. By J. G. SWAYNE, M.D. ... ... ... ... ... ... 170

CLINICAL RECORDS:

Chronic Pharyngitis with Elongated Uvula. By P. WATSON WILLIAMS, M.B. Lond. ... ... ... ... .. ... ... ... ... ... ... ... ... 189

Tubercle of Medulla Oblongata. By J. MICHELL CLARKE, M.B., M.A. 194

HEALTH-RESORTS:

Weston-super-Mare. By CHARLES VERNON HITCHINS ... ... ... ... ... 198

REVIEWS OF BOOKS ... ... ... ... ... ... ... ... ... ... ... ... ... ... 207

THE INTERNATIONAL MEDICAL CONGRESS ... ... ... ... ... ... ... ... 214

NOTES ON PREPARATIONS FOR THE SICK ... ... ... ... ... ... ... ... 215

PERISCOPE ... ... ... ... ... ... ... ... ... ... ... ... ... ... ... 217

SCRAPS ... ... ... ... ... ... ... ... ... ... ... ... ... ... ... ... 223

